# Association Between Socioeconomic Inequalities in Pain and All-Cause Mortality in the China Health and Retirement Longitudinal Study: Longitudinal Cohort Study

**DOI:** 10.2196/54309

**Published:** 2024-07-12

**Authors:** Zhuo Zhang, Dongmei Xue, Ying Bian

**Affiliations:** 1 Institute of Chinese Medical Sciences State Key Laboratory of Quality Research in Chinese Medicine University of Macau Taipa Macao

**Keywords:** pain, equality, all-cause mortality, concentration index, decomposition

## Abstract

**Background:**

Few studies focus on the equality of pain, and the relationship between pain and death is inconclusive. Investigating the distribution of pain and potential mortality risks is crucial for ameliorating painful conditions and devising targeted intervention measures.

**Objective:**

Our study aimed to investigate the association between inequalities in pain and all-cause mortality in China.

**Methods:**

Longitudinal cohort data from waves 1 and 2 of the China Health and Retirement Longitudinal Study (2011-2013) were used in this study. Pain was self-reported at baseline, and death information was obtained from the 2013 follow-up survey. The concentration index and its decomposition were used to explain the inequality of pain, and the association between pain and death was analyzed with a Cox proportional risk model.

**Results:**

A total of 16,747 participants were included, with an average age of 59.57 (SD 9.82) years. The prevalence of pain was 32.54% (8196/16,747). Among participants with pain, the main pain type was moderate pain (1973/5426, 36.36%), and the common pain locations were the waist (3232/16,747, 19.3%), legs (2476/16,747, 14.78%) and head (2250/16,747, 13.44%). We found that the prevalence of pain was concentrated in participants with low economic status (concentration index –0.066, 95% CI –0.078 to –0.054). Educational level (36.49%), location (36.87%), and economic status (25.05%) contributed significantly to the inequality of pain. In addition, Cox regression showed that pain was associated with an increased risk of all-cause mortality (hazard ratio 1.30, 95% CI 1.06-1.61).

**Conclusions:**

The prevalence of pain in Chinese adults is concentrated among participants with low economic status, and pain increases the risk of all-cause death. Our results highlight the importance of socioeconomic factors in reducing deaths due to pain inequalities by implementing targeted interventions.

## Introduction

Body pain is both a sensory and emotional experience linked to actual or potential tissue damage, incorporating physiological as well as emotional and psychological dimensions [1]. Pain is now recognized as an independent pathological condition, not merely a symptom of other diseases. The biopsychosocial framework for understanding pain emphasizes the complex and interactive relationships between biological, psychological, and social determinants. Pain creates a heavy burden of disease, with chronic pain being the predominant type [2]. According to data from the 2021 US National Health Interview Survey, approximately 51.6 million US adults (20.9%) have chronic pain and 17.1 million (6.9%) experience high-impact chronic pain [3]. This scenario not only escalates the demand for health care resources and medical expenditures but also leads to diminished work efficiency and productivity loss, thereby exacerbating the economic burden. There is a significant correlation between pain and psychological health disorders, such as anxiety and depression, which further impacts the quality of life of patients [4]. Family members also experience additional pressures due to the caregiving responsibilities for individuals with pain. The management of pain, especially chronic pain, often involves the use of opioid medications, which increases the risk of drug dependency [5]. Notably, considering factors such as time, economic conditions, and pain perception, many middle-aged and older adults choose self-management strategies, including medication, acupuncture, and massage, to alleviate pain. Research also shows a significant rise in pain incidence in the last years of life, climbing from 26% to 46% [6].

The global trend of population aging is becoming increasingly significant. With advancing age, the issues of chronic musculoskeletal joint pain, neuropathic pain, vascular-related pain, and pain triggered by psychological factors are gradually intensifying. Research on the factors influencing pain and its treatment behavior in the older population in the northeast region of China indicates that the prevalence of physical pain is as high as 32.5% among people aged 60 years or older [7]. The Global Burden of Disease study also revealed that chronic pain ranks first among high-risk factors for nonlethal health impairments [8]. In the 2019 Global Burden of Disease study, it was further revealed that migraine ranks second among factors causing disabilities, and among women younger than 50 years, migraine becomes the leading cause of disability [9]. Each year, more than 10 million new cases of malignant tumors occur globally, with 30% to 60% of patients experiencing varying degrees of pain. In late-stage patients, the prevalence of pain can even reach up to 90%. Cancer pain, as one of the major complications of malignant tumors, not only greatly affects the quality of life of patients but also becomes one of the primary reasons for seeking medical attention. Healthy People 2030 emphasizes assisting individuals with high-impact chronic pain in managing their pain safely and reducing its effects [10]. Consequently, cancer pain control has been listed as one of the 4 key priorities in the World Health Organization’s cancer treatment planning [11].

Investigating the distribution of pain is crucial for ameliorating painful conditions and devising targeted intervention measures. Previous research has predominantly focused on the unequal distribution of pain across gender, race, and disease management. This is chiefly due to the understanding of pain as an issue intricately connected to fairness and justice, set against a societal framework marked by discrimination and structural violence [12]. However, there has been relatively scant attention given to studies investigating the distribution of pain among different socioeconomic status (SES) groups, especially in developing countries. Socioeconomic factors encompass indicators such as education, occupation, economic status, and social support. The association between socioeconomic factors and chronic diseases has been reported in numerous studies, linking lower SES to higher prevalence and severity of pain. However, results pertaining to the association between socioeconomic factors and pain are sometimes contradictory, potentially due to the variables chosen for SES, the methods used, and the procedures implemented. Macro data indicates that the prevalence of any type of pain in lower- and middle-income countries is twice that in high-income countries. Mortality rates may be influenced by pain, as evidenced by a cohort study and meta-analysis comprising over 500,000 research participants, which found that individuals with chronic widespread pain have a higher mortality rate [13]. On the other hand, some studies have arrived at opposite conclusions. A meta-analysis revealed only a moderate and nonsignificant association between chronic pain and all-cause mortality, with the degree of association being slightly stronger for widespread pain, though the combined estimate still remained nonsignificant [14].

Despite previous studies analyzing the relationship between pain and all-cause mortality, research focusing on the inequality of pain and its relation to all-cause mortality rates is scarce, particularly in developing countries. Moreover, even in large cohorts, results are not entirely consistent, necessitating further research. Therefore, we have used data from the China Health and Retirement Longitudinal Study (CHARLS) survey to quantify the association between pain in Chinese adults and all-cause mortality, and additionally, we have explored the unequal conditions of pain.

## Methods

### Data Sources and Study Population

The data used in this study came from CHARLS, which conducted a national baseline survey in 2011 and followed up in 2013, 2015, and 2018. The project conducted multistage sampling in 150 counties and 450 communities (villages) in 28 provinces (autonomous regions and municipalities directly under the central government), and conducted questionnaire surveys and face-to-face interviews with middle-aged and older individuals (aged ≥45 years) and their families, aiming to analyze the current situation of aging in China and promote high-quality research to solve problems related to aging. This study used baseline data from 2011 (wave 1), excluding participants with missing information on pain, economic status, and sociodemographic characteristics (n=469) and those aged <45 years (n=492); a total of 16,747 participants were included. Considering that data were available for interview status (ie, indicating that the participant was either alive or dead) and exact time of death in 2013 (wave 2), we used the 2013 data to account for all-cause deaths; a total of 394 all-cause deaths were followed. The screening flow chart is shown in Multimedia Appendix 1, Figure S1.

### Pain, Economic Status, and All-Cause Mortality

Pain was the primary independent variable in this study. Pain was measured using a questionnaire that asked participants whether they had pain, as well as the severity and location of the pain. This study used the CHARLS 2011 question “Are you often troubled with any body pains?” to determine whether the participants had pain problems and the question “How bad is your pain?” to determine the severity of pain. The response options included mild, moderate, and severe. Moreover, the study used the question “In what part of your body do you feel pain?” to determine the location of pain, including head, shoulders, arms, wrists, fingers, chest, stomach, back, waist, buttocks, legs, knees, ankles, toes, and neck. In the Constructed Expenditure, Income, and Wealth Database published by CHARLS in 2017, the indictor *IM_PCE* was used for economic status. All-cause death was the primary outcome of the study, and information on deaths came from the 2013 survey. There were no secondary outcome events, and follow-up was terminated when an outcome event occurred.

### Covariates

We defined covariates using information collected from the baseline survey. The covariates and grouping in this study included age (45-60, 60-75, and ≥75 years), gender (male and female), education level (illiterate, primary school, secondary/high school, and university or above), marital status (never married; married; and separated, divorced, or widowed), smoking status (nonsmokers and current smokers), drinking status (never, occasionally, and regularly), BMI (<25 kg/m^2^, 25-30 kg/m^2^, and ≥30 kg/m^2^), and location (rural village or urban community). BMI was calculated by dividing weight in kilograms by the square of height in meters (kg/m^2^).

### Statistical Analysis

This study describes the pain status and sociodemographic characteristics of the participants. Using CHARLS sampling weights (*ind_weight_ad2*), the data were extrapolated to estimate the percentage of Chinese adults aged ≥45 years experiencing pain. Continuous variables are represented as the mean and SD, and a 2-tailed *t* test was used for comparisons between groups. Categorical variables were represented as numbers and percentages, and comparisons between groups were performed using the *χ*^2^ test. Logistic regression was used to describe the impact of sociodemographic characteristics on pain. Second, the concentration index was used to analyze the equality of pain, and the economic status indicator was per capita expenditure. The concentration index was defined as twice the area between the curve and the diagonal, ranging from –1 to 1. A positive concentration index value indicated that the disease distribution showed a trend to occur among groups with higher economic status, while a negative value indicated the opposite. The closer the concentration index was to 0, the more equal was the disease distribution [15]. Decomposition analysis was further used to describe the contribution of each factor to inequality. The reliability of using the concentration index and its decomposition to measure pain inequalities has been well established in previous studies [16]. Finally, a Cox proportional hazards regression model was used to explore the relationship between pain and all-cause death, and further stratified analysis was conducted based on sociodemographic characteristics. All reported *P* values were 2-sided, and statistical analyses were conducted with Stata (version 16.0; StataCorp).

The concentration index was calculated in detail as follows:




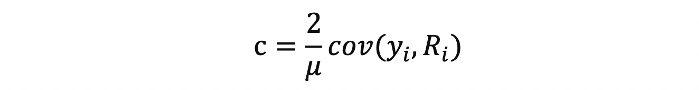




In the formula, *R_i_* denotes the proportion of individuals ranked by SES, *y_i_* denotes the prevalence of pain, and *μ* denotes the average prevalence of pain.

The decomposition method for the concentration index allowed decomposing the index into contributions from various influencing factors. The associated linear regression model is specified below:




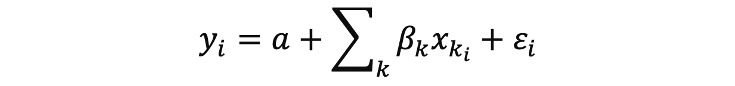




In the formula, *β_k_* denotes the regression coefficient. The concentration index was decomposed using the equation provided below:




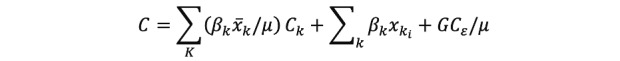




In the formula, *C_k_*, *β_k_*, and 
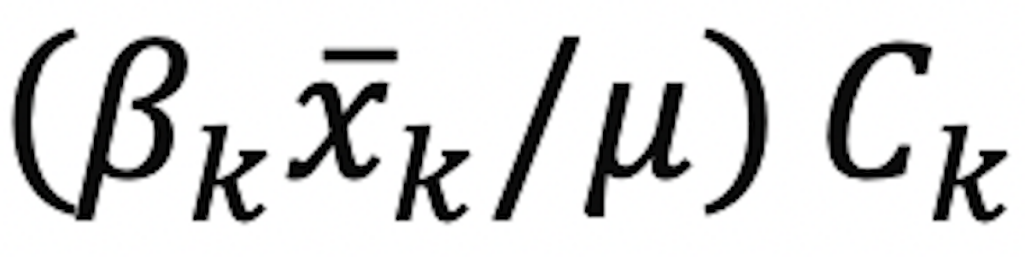
 denote the concentration index, regression coefficient, and contribution, respectively. 

### Ethical Considerations

All practices involving human participants in this study were conducted in strict adherence to the principles of the Declaration of Helsinki. Ethical clearance for the initial CHARLS data collection was obtained from the Peking University Ethical Review Committee (IRB00001052-11015). Moreover, every participant in the CHARLS cohort willingly provided their written informed consent, ensuring ethical compliance and participant awareness. The present analysis received approval from the University of Macau’s research ethics subpanel committee (BSERE21-APP012-ICMS). All respondents signed informed consent forms, and the project was reviewed by the Institutional Review Board of Peking University.

## Results

### Pain Prevalence and Sociodemographic Characteristics of the Participants

A total of 16,747 participants were included in the study, with an average age of 59.57 (SD 9.82) years, of whom 48.94% (8196/16,747) were male. A total of 31% (95% CI 29.36-32.69) of participants had pain, of whom 36.36% (1973/5426) had moderate pain. The top 3 pain locations were the waist (3232/16,747, 19.3%), legs (2476/16,747, 14.78%), and head (2250/16,747, 13.44%). There were differences in pain status among different groups by age, gender, education status, marital status, smoking, alcohol consumption, economic status, and location (Table 1). Female gender and regular drinking increased the risk of pain. Higher education, higher economic status, and living in an urban community reduced the risk of pain (Multimedia Appendix 1, Table S1).

**Table 1:** Sample characteristics of adults aged ≥45 years from China Health and Retirement Longitudinal Study by pain status.

Baseline characteristics		Total (N=16,747)	No pain (n=11,298)	Pain (n=5449)	*P* value	
Age (years), mean (SD)		59.57 (9.82)	59.29 (9.86)	60.17 (9.71)	<.001	
**Age groups (years), n (%)**						<.001
	45-60	9545 (57)	6587 (58.3)	2958 (54.29)		
	60-75	5789 (34.57)	3774 (33.4)	2015 (36.98)		
	≥75	1413 (8.44)	937 (8.29)	476 (8.74)		
**Gender**						<.001
	Male	8196 (48.94)	6042 (53.48)	2154 (39.53)		
	Female	8551 (51.06)	5256 (46.52)	3295 (60.47)		
**Education level**						<.001
	Illiterate	4631 (27.65)	2750 (24.34)	1881 (34.52)		
	Primary school	6574 (39.25)	4239 (37.52)	2335 (42.85)		
	Secondary/high school	5146 (30.73)	3961 (35.06)	1185 (21.75)		
	University or above	396 (2.36)	348 (3.08)	48 (0.88)		
**Marital status**						<.001
	Never married	156 (0.93)	107 (0.95)	49 (0.9)		
	Married	14,567 (86.98)	9929 (87.88)	4638 (85.12)		
	Separated, divorced, or widowed	2024 (12.09)	1262 (11.17)	762 (13.98)		
**Smoking status**						<.001
	Nonsmokers	11,377 (67.93)	7458 (66.01)	3919 (71.92)		
	Current smokers	5370 (32.07)	3840 (33.99)	1530 (28.08)		
**Drinking status**						<.001
	Never	9779 (58.39)	6406 (56.7)	3373 (61.9)		
	Occasionally	1705 (10.18)	1187 (10.51)	518 (9.51)		
	Regularly	5263 (31.43)	3705 (32.79)	1558 (28.59)		
**BMI (kg/m^2^)**						.20
	<25	12,701 (75.84)	8613 (76.23)	4088 (75.02)		
	25-30	3372 (20.13)	2232 (19.76)	1140 (20.92)		
	≥30	674 (4.02)	453 (4.01)	221 (4.06)		
**Economic status**						<.001
	Tertile 1	5583 (33.34)	3556 (31.47)	2027 (37.2)		
	Tertile 2	5583 (33.34)	3648 (32.29)	1935 (35.51)		
	Tertile 3	5581 (33.33)	4094 (36.24)	1487 (27.29)		
**Location**						<.001
	Rural village	12,888 (76.96)	8291 (73.38)	4597 (84.36)		
	Urban community	3859 (23.04)	3007 (26.62)	852 (15.64)		
**Severity of pain**						—^a^
	Mild	1358 (25.03)	—	1358 (25.03)		
	Moderate	1973 (36.36)	—	1973 (36.36)		
	Severe	2095 (38.61)	—	2095 (38.61)		
**Location of pain**						—
	Head	2250 (13.44)	—	2250 (13.44)		
	Shoulders	2038 (12.17)	—	2038 (12.17)		
	Arms	1655 (9.88)	—	1655 (9.88)		
	Wrists	1057 (6.31)	—	1057 (6.31)		
	Fingers	990 (5.91)	—	990 (5.91)		
	Chest	1068 (6.38)	—	1068 (6.38)		
	Stomach	1436 (8.57)	—	1436 (8.57)		
	Back	1533 (9.15)	—	1533 (9.15)		
	Waist	3232 (19.3)	—	3232 (19.3)		
	Buttocks	633 (3.78)	—	633 (3.78)		
	Legs	2476 (14.78)	—	2476 (14.78)		
	Knees	2178 (13.01)	—	2178 (13.01)		
	Ankles	960 (5.73)	—	960 (5.73)		
	Toes	563 (3.36)	—	563 (3.36)		
	Neck	1135 (6.78)	—	1135 (6.78)		

^a^Not applicable.

### Concentration and Its Decomposition

**Figure 1:**
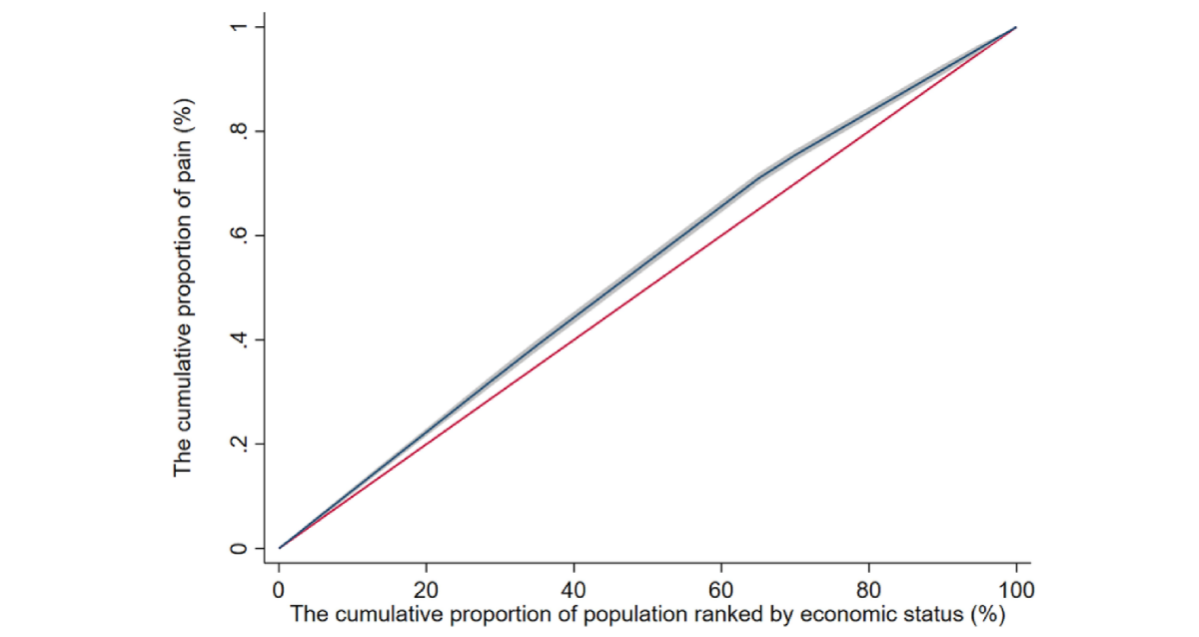
Concentration curve of pain among Chinese adults aged ≥45 years from the China Health and Retirement Longitudinal Study, 2011-2012. The shadow around the curve represents the 95% CI.

The concentration index of pain among Chinese adults was –0.066 (95% CI –0.078 to –0.054), which indicates that the prevalence of pain was higher among those with low economic status (Figure 1). Decomposition analysis found that old age, female gender, lower educational level, never being married or having another marriage status, living in a rural community, smoking, and having a lower economic status increased the inequality of pain and never or sometimes drinking and lower BMI decreased the inequality of pain. Among these factors, educational level (36.49%), location (36.87%), and economic status (25.05%) contributed significantly to the inequality of pain (Table 2). Further analysis showed that pain severity and the different parts of the body affected by pain (head, shoulders, arms, wrists, fingers, chest, stomach, back, waist, buttocks, legs, knees, ankles, toes, and neck) were concentrated in participants with low economic status, and the legs exhibited the most severe inequality (concentration index –0.121, 95% CI –0.141 to –0.101; Multimedia Appendix 1, Table S2).

**Table 2:** Decomposition analysis on the inequality of pain among Chinese adults aged ≥45 years.

Variables and categories		Elasticity	Concentration index	Contribution		Contribution rate (%)	
**Age, (years)**							0.94
	60-75	0.077	–0.075		–0.006		
	≥75	–0.018	–0.123		0.002		
Female gender		0.890	–0.004	–0.003		0.9	
**Educational level**							36.49
	Illiteracy	0.803	–0.204		–0.164		
	Primary school	1.105	–0.055		–0.061		
	Secondary/high school	0.439	0.202		0.088		
**Marriage status**							0.38
	Never married	0.001	–0.178		0.000		
	Separated, divorced, or widowed	0.037	–0.036		–0.001		
Smoker		0.031	–0.016	–0.001		0.14	
**Drinking status**							–0.42
	Never	–0.256	–0.008		0.002		
	Occasionally	–0.026	0.019		0.000		
**BMI (kg/m^2^)**							–0.35
	<25	0.026	–0.014		0.000		
	25-30	0.045	0.037		0.002		
**Economic status**							25.05
	Tertile 1	0.141	–0.667		–0.094		
	Tertile 2	0.162	0.000		0.000		
Location: rural village		1.073	–0.129	–0.138		36.87	

### Association of Pain With Risks of All-Cause Mortality

**Figure 2:**
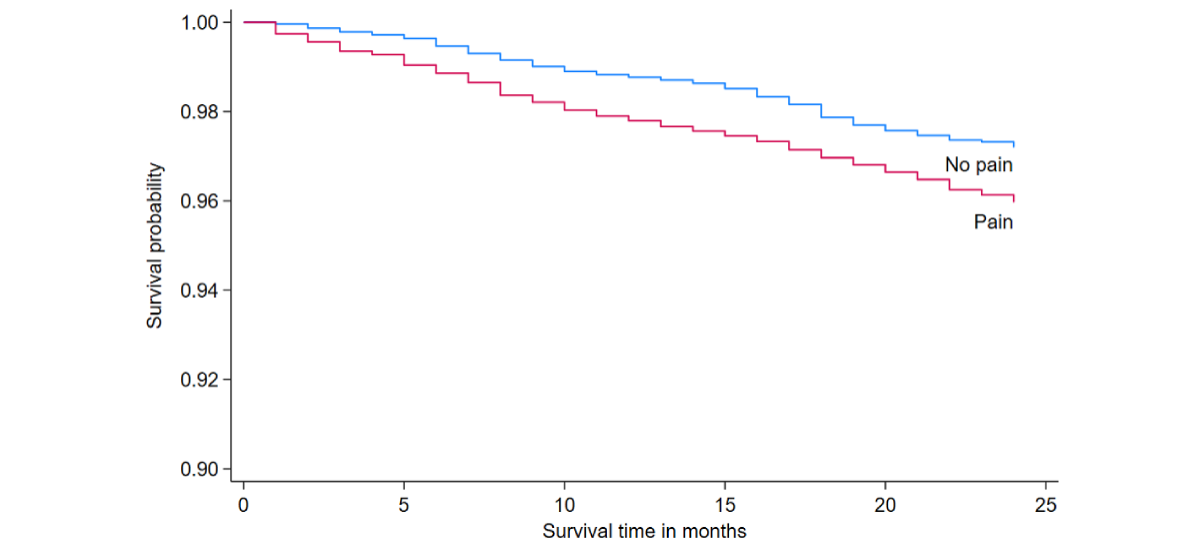
Kaplan-Meier curves for the cumulative risk of all-cause mortality by pain status.

**Figure 3:**
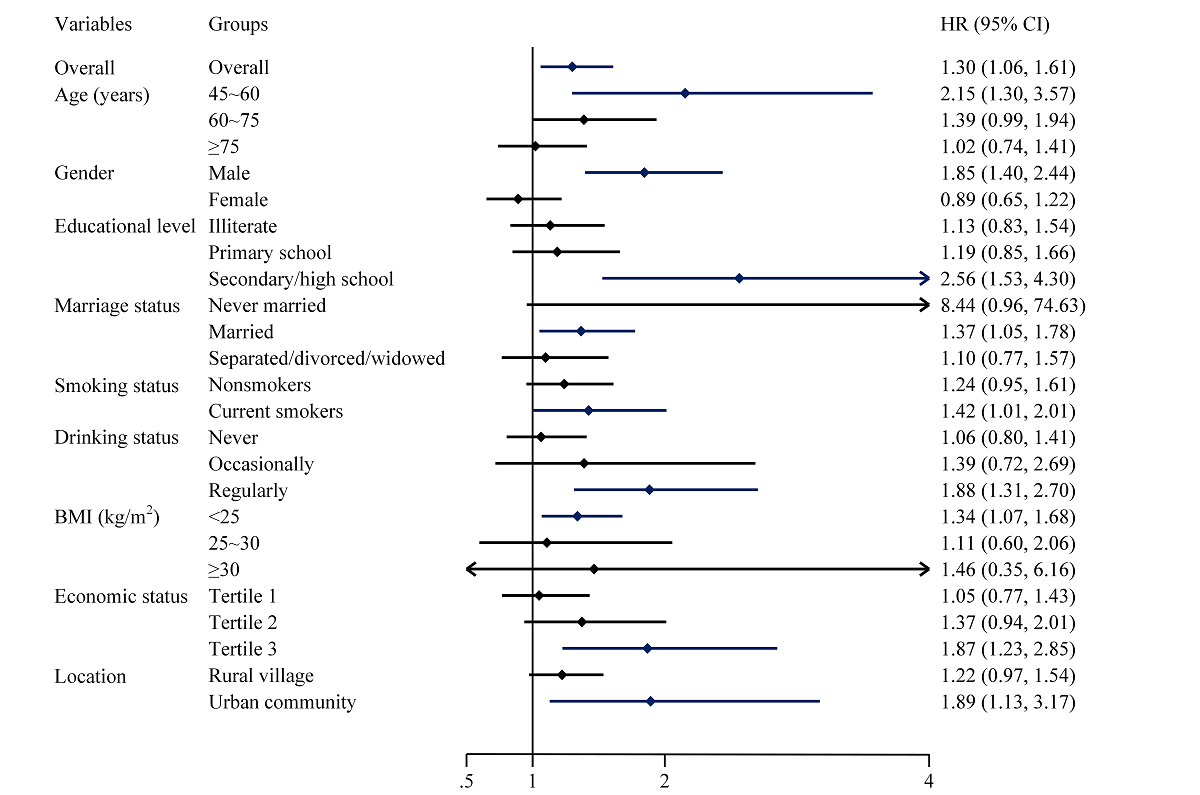
Association of pain with risk of all-cause mortality among Chinese adults aged ≥45 years from 2011 to 2012. Age, gender, education level, marriage status, location, smoking status, drinking status, BMI, and economic status were adjusted in addition to the subgroup variables. Subgroup analysis for the “university or above” educational level was not performed due to the limited sample size. HR: hazard ratio.

After 3 years of follow-up, 394 death events were reported, for a mortality rate of 2.35%. Participants with pain had a higher mortality rate than those without pain (log-rank test; *P*=.002) (Figure 2). After adjusting for all covariates, Cox regression found that pain was associated with an increased risk of all-cause mortality (hazard ratio [HR] 1.30, 95% CI 1.06-1.61; Figure 3). Severity of pain also increased the risk of all-cause mortality (moderate: HR 1.36, 95% CI 1.02-1.83; severe: HR 1.42, 95% CI 1.07-1.87). The analysis focusing on specific types of pain showed that chest pain was significantly associated with all-cause mortality (Multimedia Appendix 1, Table S3). In addition, we performed stratified analyses according to sociodemographic characteristics, and the results for each group differed slightly from the overall results, but the different groups showed a trend that pain was associated with a higher risk of all-cause mortality across different groups (Figure 3).

## Discussion

### Principal Results

This study found that there was inequality in the prevalence of pain in nationally representative data from CHARLS. This inequality was concentrated in people with lower economic conditions. Education level, location, and economic status contributed significantly to this inequality. We further found that pain increased the risk of all-cause mortality. These findings outline the current inequitable status of pain and potential mortality risks in China.

### Comparison With Prior Work

Previous studies have revealed correlations between pain and economic status. A meta-analysis found that compared to people with high SES, people with low and medium SES had an increased risk of pain [16]. We reached similar conclusions using the concentration index. In addition, studies on the association between SES and pain have been mainly concentrated in high-income countries, with fewer prospective studies conducted in low- and middle-income countries, and our results make up for this deficiency. In addition, we found that economic status, education level, and location were associated with greater pain inequality. All 3 variables are common indicators of SES [17-19]. Individuals with lower SES tend to report higher prevalence of pain [20,21]. Individuals with low SES often face barriers to accessing medical resources, including appropriate resources for pain management [19]. Limited access to medical resources may lead to delayed diagnosis and treatment of pain symptoms, exacerbating pain. Also, lower SES is associated with heavy physical work or a sedentary lifestyle [22,23]. These characteristics may contribute to the development of musculoskeletal pain. Chronic exposure to economic stress, including unemployment, housing instability, and food insecurity, could lead to chronic psychosocial stress. This stress is associated with the development of pain, involving the hypothalamic-pituitary-adrenal axis and neuroinflammatory status [16,24].

Pain is a sensory and emotional experience with great biological, psychological, and sociological complexity, which not only seriously affects the quality of life, but may also adversely affect longevity [1,25,26]. Song and Chung [27] used data from the Korean Longitudinal Study of Aging (2006-2016) and found there was a statistically significant relationship between pain and mortality risk, and this risk increased with the intensity of pain. Smith et al [28] also found this association in the English Longitudinal Study of Ageing. Our research led to similar conclusions. There have also been studies reporting an association between different pain sites and death. A systematic review and meta-analysis of cohort study data found that back pain was associated with increased all-cause mortality in women and people with more severe back pain [29]. Cleveland et al [30] found that knee pain was associated with increased mortality in a community-based cohort study. However, it is hard to say whether pain directly increases the risk of death. Chen et al [31], using data from the UK Biobank, found that at least half of the association between chronic musculoskeletal pain and increased all-cause mortality may be mediated by 4 factors: physical activity, smoking status, alcohol consumption, and opioid use. The effect of pain on all-cause death is a complex issue and more research is needed to further understand this association. Current evidence highlights the importance of pain management and effective pain management strategies, including comprehensive treatments and attention to the patient’s lifestyle and mental health, which may help reduce the adverse health outcomes associated with pain [32-36]. With the increase of aging in China, it is necessary to accurately understand pain and conduct policy interventions for pain.

### Limitations

Our findings highlight the importance of equality-oriented policies for pain to reduce deaths associated with pain inequality. However, our study has some potential limitations. First, information about pain, its severity, and its location were self-reported, which could lead to recall bias. Second, the data for estimating pain equality in this study were from 2011, which may introduce bias relative to the current situation. Third, some studies have found that new-onset pain may significantly increase the risk of death [27], and our study did not consider the duration of pain, which led to insufficiently detailed results. Fourth, some factors, such as recent injuries, may influence the association between pain and all-cause mortality. However, CHARLS did not investigate this, potentially leading to an overestimation of the effect of pain on all-cause mortality. Finally, the study participants were aged 45 to 75 years, which limits our estimation of the equality of pain prevalence in people older than 75 years. Nevertheless, our study has important implications for highlighting the impact of pain on health.

### Conclusion

The prevalence of pain in Chinese adults was concentrated among participants with low economic status, and pain increased the risk of all-cause death. Our results highlight the importance of socioeconomic factors in reducing deaths due to pain inequality by implementing targeted interventions to reduce pain inequality.

## Appendix

Multimedia Appendix 1 Tables S1-S3 and Figure S1.

## Abbreviations

CHARLS: China Health and Retirement Longitudinal Study
HR: hazard ratio
SES: socioeconomic status

## Data Availability

All data sets are accessible through the China Health and Retirement Longitudinal Study database [37]. Researchers interested in using these data can register for an account on the website.
