# Supplementary material for: Association Between Socioeconomic Inequalities in Pain and All-Cause Mortality in the China Health and Retirement Longitudinal Study: Longitudinal Cohort Study
Source: JMIR Public Health Surveill. 2024 Jul 12;10:e54309. doi: 10.2196/54309 (PMC11282390; doi:10.2196/54309)
Supplement: Multimedia Appendix 1 [file publichealth_v10i1e54309_app1.docx]

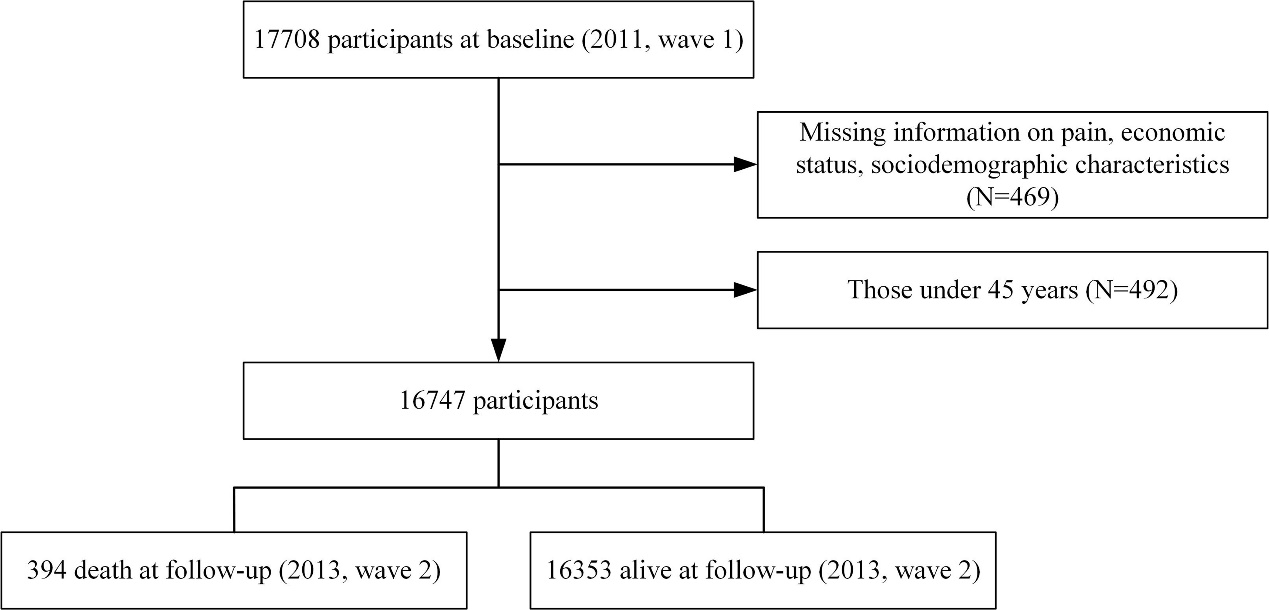


Figure S1. Flow diagram of participants selection.

Table S1. Logistic regression showing association between baseline characteristics and pain among Chinese adults aged ≥45 years.

| Baseline characteristics | OR (95%CI) | P |
| --- | --- | --- |
| Age (years) |  |  |
| 60~75 | 1.08 (1.00, 1.16) | 0.056 |
| ≥75 | 0.93 (0.81, 1.07) | 0.300 |
| Gender |  |  |
| Female | 1.76 (1.60, 1.94) | ＜0.001 |
| Education level |  |  |
| Primary school | 0.97 (0.89, 1.06) | 0.503 |
| Secondary/high school | 0.62 (0.56, 0.69) | ＜0.001 |
| university or above | 0.39 (0.28, 0.54) | ＜0.001 |
| Marital status |  |  |
| Married | 0.98 (0.69, 1.38) | 0.903 |
| Separated/divorced/widowed | 1.08 (0.76, 1.55) | 0.666 |
| Smoking status |  |  |
| Current smokers | 1.03 (0.94, 1.13) | 0.486 |
| Drinking status |  |  |
| Occasionally | 1.06 (0.94, 1.19) | 0.316 |
| Regularly | 1.15 (1.06, 1.26) | 0.002 |
| BMI (kg/㎡) |  |  |
| 25~30 | 1.06 (0.98, 1.16) | 0.149 |
| ≥30 | 0.99 (0.83, 1.17) | 0.899 |
| Economic status |  |  |
| Quartile 2 | 1.02 (0.94, 1.11) | 0.611 |
| Quartile 3 | 0.87 (0.80, 0.95) | 0.003 |
| Location |  |  |
| Urban community | 0.64 (0.58, 0.70) | ＜0.001 |

Table S2. Concentration index of pain of Chinese adults aged ≥45 years according to severity and part.

| Pain characteristics | concentration index (95%CI) | P |
| --- | --- | --- |
| Severity of pain^a^ | -0.079 (-0.260, -0.092) | <0.001 |
| Part of pain |  |  |
| Head | -0.102 (-0.122, -0.081) | <0.001 |
| Shoulder | -0.070 (-0.092, -0.047) | <0.001 |
| Arm | -0.089 (-0.114, -0.064) | <0.001 |
| Wrist | -0.109 (-0.141, -0.077) | <0.001 |
| Fingers | -0.104 (-0.137, -0.071) | <0.001 |
| Chest | -0.099 (-0.131, -0.068) | <0.001 |
| Stomach | -0.090 (-0.116, -0.063) | <0.001 |
| Back | -0.099 (-0.125, -0.073) | <0.001 |
| Waist | -0.078 (-0.095, -0.061) | <0.001 |
| Buttocks | -0.094 (-0.135, -0.052) | <0.001 |
| Leg | -0.121 (-0.141, -0.101) | <0.001 |
| Knees | -0.104 (-0.126, -0.083) | <0.001 |
| Ankle | -0.087 (-0.120, -0.053) | <0.001 |
| Toes | -0.102 (-0.146, -0.058) | <0.001 |
| Neck | -0.080 (-0.110, -0.049) | <0.001 |

^a^ Severity of pain was divided into no pain, mild, moderate and severe.

Table S3. Association of pain characteristics with risk of all-cause mortality.

| Pain characteristics | HR (95%CI) | P |
| --- | --- | --- |
| Severity of pain ^a^ |  |  |
| Mild | 1.00 (0.67, 1.49) | 0.993 |
| Moderate | 1.36 (1.02, 1.83) | 0.038 |
| Severe | 1.42 (1.07, 1.87) | 0.014 |
| Part of pain |  |  |
| Head | 1.10 (0.83, 1.47) | 0.503 |
| Shoulder | 1.07 (0.79, 1.45) | 0.649 |
| Arm | 1.17 (0.86, 1.60) | 0.318 |
| Wrist | 1.09 (0.73, 1.62) | 0.685 |
| Fingers | 1.40 (0.97, 2.02) | 0.071 |
| Chest | 1.55 (1.12, 2.16) | 0.008 |
| Stomach | 1.01 (0.70, 1.44) | 0.969 |
| Back | 1.15 (0.83, 1.59) | 0.404 |
| Waist | 1.08 (0.84, 1.38) | 0.565 |
| Buttocks | 1.24 (0.79, 1.95) | 0.355 |
| Leg | 1.23 (0.95, 1.58) | 0.115 |
| Knees | 0.91 (0.68, 1.23) | 0.550 |
| Ankle | 1.00 (0.66, 1.52) | 0.991 |
| Toes | 0.94 (0.56, 1.57) | 0.804 |
| Neck | 0.88 (0.58, 1.35) | 0.565 |

^a^ The reference group was no pain.
